# Face Recognition System for Set-Top Box-Based Intelligent TV

**DOI:** 10.3390/s141121726

**Published:** 2014-11-18

**Authors:** Won Oh Lee, Yeong Gon Kim, Hyung Gil Hong, Kang Ryoung Park

**Affiliations:** Division of Electronics and Electrical Engineering, Dongguk University, 26 Pil-dong 3-ga, Jung-gu, Seoul 100-715, Korea; E-Mails: 215p8@hanmail.net (W.O.L.); csokyg@dongguk.edu (Y.G.K.); hell@dongguk.edu (H.G.H.)

**Keywords:** set-top box, face recognition, in-plane rotation, multi-level local binary pattern

## Abstract

Despite the prevalence of smart TVs, many consumers continue to use conventional TVs with supplementary set-top boxes (STBs) because of the high cost of smart TVs. However, because the processing power of a STB is quite low, the smart TV functionalities that can be implemented in a STB are very limited. Because of this, negligible research has been conducted regarding face recognition for conventional TVs with supplementary STBs, even though many such studies have been conducted with smart TVs. In terms of camera sensors, previous face recognition systems have used high-resolution cameras, cameras with high magnification zoom lenses, or camera systems with panning and tilting devices that can be used for face recognition from various positions. However, these cameras and devices cannot be used in intelligent TV environments because of limitations related to size and cost, and only small, low cost web-cameras can be used. The resulting face recognition performance is degraded because of the limited resolution and quality levels of the images. Therefore, we propose a new face recognition system for intelligent TVs in order to overcome the limitations associated with low resource set-top box and low cost web-cameras. We implement the face recognition system using a software algorithm that does not require special devices or cameras. Our research has the following four novelties: first, the candidate regions in a viewer's face are detected in an image captured by a camera connected to the STB via low processing background subtraction and face color filtering; second, the detected candidate regions of face are transmitted to a server that has high processing power in order to detect face regions accurately; third, in-plane rotations of the face regions are compensated based on similarities between the left and right half sub-regions of the face regions; fourth, various poses of the viewer's face region are identified using five templates obtained during the initial user registration stage and multi-level local binary pattern matching. Experimental results indicate that the recall; precision; and genuine acceptance rate were about 95.7%; 96.2%; and 90.2%, respectively.

## 1. Introduction

In recent times, the broadcasting environment has changed significantly owing to the prevalence of digital TVs, internet protocol (IP) TVs, and smart TVs that provide a variety of multimedia services and multiple channels. Consequently, audiences can access their desired amount of multimedia content. Due to these developments in the broadcasting environment, many broadcasters, advertising agents, media agents, and audience rating survey companies are increasingly interested in measuring the viewer's watching patterns. As a result, considerable research is being focused on interactive TV [1–3]. Many intelligent TVs include cameras that facilitate face recognition technologies and can be used to identify viewers and provide personalized services [3–7]. Zuo *et al.* presented a consumer-oriented face recognition system called *HomeFace*. Their system can be embedded in a smart home environment that includes a smart TV for user identification [3]. It uses skin-color-based, geometry-based, and neural-network (NN)-based face detectors to detect face regions. In addition, it uses multi-stage, rejection-based linear discriminant analysis (LDA) to classify faces. However, their system cannot recognize faces that are in in-plane or out-of-plane rotation states or those that are captured at low resolutions. In-plane rotation of a face frequently occurs when a viewer watches the TV while lying on his or her side. An *et al.*, proposed a real-time face analysis system that can detect and recognize human faces and their expressions using adaptive boosting (Adaboost) LDA (Ada-LDA), and multi-scale and multi-position local binary pattern matching (MspLBP) [4]. However, their system cannot analyze faces with in-plane rotation. Lee *et al.*, proposed a smart TV interaction system that performs face detection and classification based on uniform local binary patterns (ULBPs) and support vector machines (SVMs). In addition, they use local Gabor binary pattern histogram sequences (LGBPHS) for face recognition [5,7]. However, their system requires an additional near-infrared camera with illuminators in order to function properly. Lin *et al.* introduced a prototype multi-facial recognition technique aided IPTV system that is called *EyeTV*. Their system uses an IP camera to acquire the facial features of users, and a multi-facial recognition technique is employed in order to recognize the viewer's identity [6]. It also stores the viewing history of a user's group automatically. However, the system does not deal with in-plane rotation of faces or faces in various poses.

We propose a new face recognition system for intelligent TVs equipped with supplementary low resource set-top boxs (STBs) that overcomes the shortcomings of the previous proposals stated above. Recognizing the faces of TV viewers is different from face recognition for access control. The in-plane rotations of faces occur frequently in cases of face recognition in a TV environment because viewers often watch TV while lying on their sides. Therefore, our system compensates for in-plane rotations of face regions by measuring the similarities between the left and right half sub-regions of the face regions. In addition, it recognizes faces in various poses based on five templates that are stored during the enrollment process and multi-level local binary patterns (MLBPs).

The remainder of this paper is organized as follows: we describe the proposed system and the method in Section 2, present experimental results in Section 3, and summarize and present concluding remarks in Section 4.

## 2. Proposed System and Method

### 2.1. Proposed System Architecture

**Figure 1.**
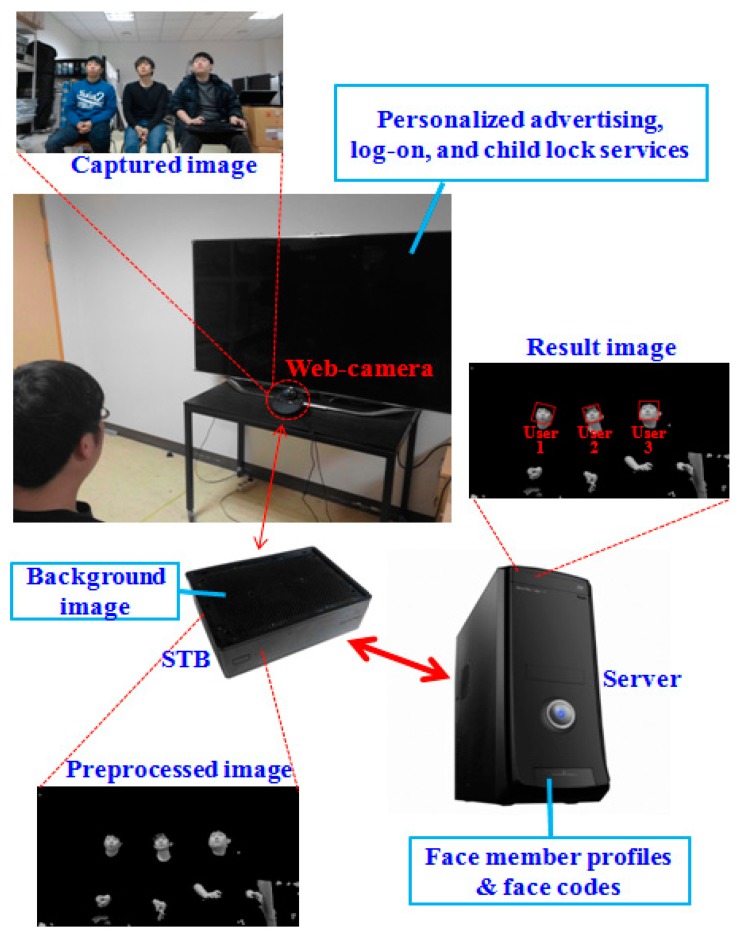
Proposed face recognition system for digital TV with supplementary STB.

Figure 1 shows the structure of our proposed face recognition system. It consists of a web-camera, an STB for preprocessing, and a server for face recognition.

The STB is connected to the web-camera and is used on the client side. A commercial web camera (Logitech BCC950 [8]) was used during the experiment. It has a CMOS image sensor (1920 × 1080 pixels) and a diagonal field of view of 78°. The data interface for this camera is USB 2.0. Owing to the processing power limitations of the STB, we capture images at a resolution of 1280 × 720 pixels.

**Figure 2.**
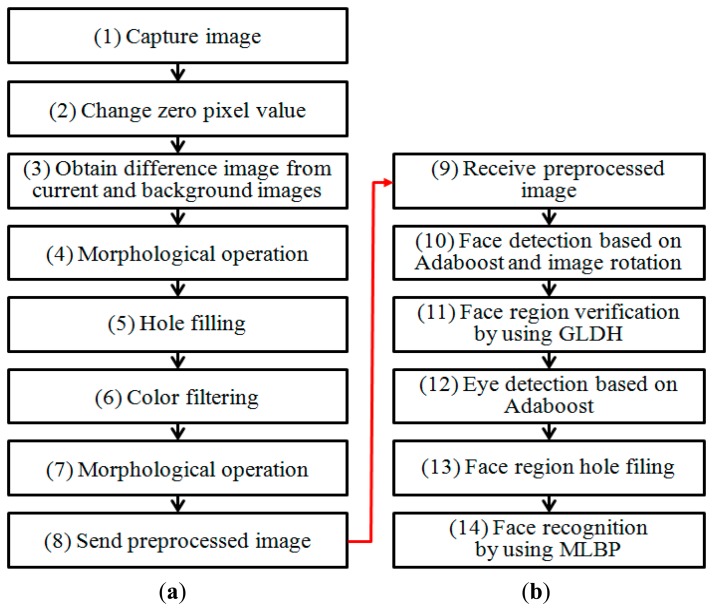
Flowchart of our proposed method: (**a**) Client part, (**b**) Server part.

The background image is also registered for preprocessing on the client side. Our system automatically recommends that the user update the background image based on measurements of changes in pixels between the initial background image without any user and the current image. An updated background image can be saved manually by pressing a button on a remote controller. However, this procedure of manual update by user is not mandatory but optional to the user (just recommendation). Even without this procedure of the manual update, our system can detect the area of user's face by color filtering in the client side and Adaboost face detector in the server side, respectively, as shown in Figure 2.

Then, preprocessing is conducted and the image of a face candidate region is sent to the server via the communication network. On the server side, family member profiles are pre-registered for face recognition. The family member profiles and their face codes are enrolled through the STB and the web-camera during the initial user registration stage. Based on this information, face recognition is performed using MLBP matching. The experimental results indicate that our proposed system can provide personalized services such as advertising, log on, and child lock.

### 2.2. Overview of Proposed Method

Figure 2 shows a flowchart of our proposed method. Figure 2a,b displays the client and server portions of our proposed method, respectively.

A red-green-blue (RGB) color image is captured by a camera connected to the STB in step (1) and the resulting gray image is obtained. In step (2), the zero pixel values of the gray image are converted to one in order to discriminate between the non-candidate regions of faces that are assigned as zero values and the zero pixel values of the input image. In other words, step (2) is performed in order to distinguish the original zero (black) pixels in the input image from the background areas assigned as zero (black) pixels through the step (3) of Figure 2. In step (3), the difference image between the captured image and the pre-saved background image is obtained in order to estimate user candidate areas. In step (4), morphological operations are performed to remove noise [9]. In step (5), a procedure is carried out in order to fill in holes in the face region. In step (6), the candidate areas for face regions are determined using skin color filtering in the face candidate regions obtained in step (5). Then, morphological operations are performed to remove noise from the face areas and a preprocessed image is obtained.

In step (8), the preprocessed image is sent to the server over the network. Although the Adaboost method has been widely used for face detection [10,11], we use the method in Figure 2 for the following reasons. The proposed face recognition system is implemented in intelligent TV with supplementary, low resource STB. The processing power of STB is considerably low and therefore, the Adaboost method can overload the STB. In addition, false detections of face regions by the Adaboost method can be minimized by discarding non-face candidate regions based on the preprocessing shown in Figure 2.

On receiving the preprocessed image, the server detects the face regions (step (10)). In order to detect the face regions when in-plane rotations have occurred, a procedure comprising image rotations and face detection using the Adaboost method is performed. The Adaboost method is based on a combination of weak classifiers [10,11]. Based on these steps, multiple face boxes can exist even for a single face. Therefore, in order to detect and select the correct face box, the gray level difference histogram (GLDH) method is utilized, as shown in step (11) [12]. The GLDH method is based on the measurement of the level of similarity between the left and right half sub-regions of the face region. In step (12), the eye regions are detected based on the Adaboost method. Information about the detected eye region is utilized in order to reject incorrectly detected face regions, and face normalization is used for face recognition. The areas of the face region with holes are then filled through interpolation in step (13), and face recognition is conducted using MLBP.

In order to reduce communication load, one preprocessed image was sent to the server when a user pressed a button on the remote controller. Therefore, it was not possible to use tracking methods, such as Kalman and particle filter, that are based on successive frames to detect faces and eyes.

### 2.3. Preprocessing on the Client Side

**Figure 3.**
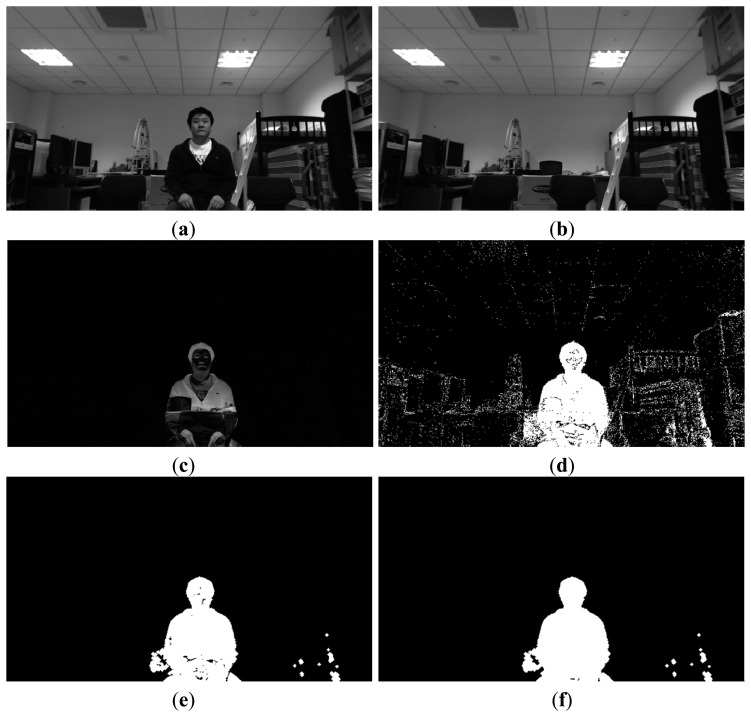
Examples illustrating segmentation of the user area: (**a**) Input image; (**b**) Background image; (**c**) Difference image obtained from (a) and (b); (**d**) Binary image of (c); (**e**) Image after morphological operation; (**f**) Image obtained by filling holes in (e).

Our proposed method is divided into the following two stages: the enrollment stage and the recognition stage. During the enrollment stage, a user inputs his/her family member code (father, mother, son, *etc.*) and a face image is captured by the web-camera. The captured image is then sent to the server after the preprocessing steps (1)–(8) (Figure 2a). The face region is then detected and facial codes are enrolled using the MLBP method, as shown in steps (9)–(14) (Figure 2b). Five face images are captured as the user gazes at the following five positions on the TV screen: top-left, top-right, center, bottom-left, and bottom-right. These images are utilized for face recognition that is robust to various facial poses. During the recognition stage, face recognition is performed based on facial codes that were saved during the enrollment stage. In general, many STBs are connected to the remote server. Thus, preprocessing of the captured image is performed by the clients in order to reduce the communication load that can arise when sending the original captured images to the server. Figure 3 illustrates the segmentation of the user area of the image—(*i.e.*, steps (1)–(5) in Figure 2).

First, an RGB image is captured and converted to a gray image (Figure 3a). The zero value pixels of the gray image are then changed to one. This is performed to distinguish between the zero value pixels in the gray image from the non-candidate regions of the faces, which are assigned zero pixel values. The difference image is obtained by subtracting the pre-saved background image from the input gray image. This image is converted into a binary image.

If the environmental illumination of the input image is different from that of the pre-saved background, the difference image can include many noisy regions in addition to the user area. In order to solve this problem, our system uses the following scheme. If there are significant pixel level differences between the initial background image without any user and the current image, our system automatically recommends that the user update the background image. The new background image can be manually saved by pressing a button on a remote controller.

Then, morphological operations such as erosion and dilation are performed in the binary image in order to remove noise. The morphological operation in Figure 2 (step (4)) is performed using the following procedures. First, an erosion operation is performed in order to remove noise and then a dilation operation is performed in order to remove small holes in face candidate regions. There are two types of possible errors in the binary image, as shown in Figure 3e. A type 1 error is defined as one in which the foreground (user area) is incorrectly identified as the background and a type 2 error is the reverse. The additional procedure to detect accurate face area is performed with the face candidate regions transmitted to the server. Thus, we designed a filter that reduces the type 1 errors by filling in holes in the face candidate regions, as defined in Equation (1):

(1) $$b(i,j)=\{\begin{array}{cc} 1, & \text{if}\frac{1}{255n^{2}}\underset{(l,m)\in B}{\text{∑}}b(i-l,j-m)\geq \alpha \\ 0, & \text{otherwise} \end{array}$$

here, *b*(*i, j*) is the binary image and *B* is the structuring elements for *n × n* pixels. We experimentally determined the values of *n* and *α* as 11 and 0.35, respectively. The *b*(*i, j*) which is satisfied with the upper condition of Equation (1) is assigned as 1 and filled as the face candidate pixel. The hole filling procedure can reduce the holes whose sizes are considerably large to be removed by the morphological operation. There are many white pixels (correctly defined as face candidate regions) around holes (incorrectly defined as non-face regions). Based on these characteristics, target pixels are converted from non-face candidate regions to face candidate ones when the proportion of white pixels is larger than the *α* threshold in the *n* × *n* mask in Equation (1).

Skin color filtering is then performed. Various color spaces can be used for estimating skin regions. They include RGB, YCrCb [13], and HSV [14]. In order to reduce the effect of brightness on color, the color spaces of YCrCb and HSV have been used for the estimation of skin color more than that of RGB. For our study, we choose the HSV color space for detecting the face color area. In general, the skin color area is defined in the HSV color space using the parameters in Equation (2) [14]:

(2) $$\begin{array}{ccc} 0{}^{\circ}\leq H\leq 50{}^{\circ}, & 0.20\leq S\leq 0.68, & 0.35\leq V\leq 1.0 \end{array}$$

Zhang *et al.* introduced a hue histogram, which they used to analyze 200 pictures of people from the Mongoloid, Caucasoid, and Negroid ethnic groups [15]. They discovered that skin color pixels are distributed mainly in the region [0°, 50°], and that there are negligible skin color pixel distributions in the region [300°, 350°]. Because more accurate face area detections can be performed based on the face candidate regions that are transmitted to the server, we use a wider range of hue values for color filtering, as defined in Equation (3), in order to reduce type 1 face detection errors. After the color filtering is performed based on Equation (3), we perform additional procedures for face detection, face region verification, eye detection, and face recognition (Figure 2b). Type 2 errors can be reduced by these additional procedures. However, if type 1 errors occur, they cannot be corrected by these additional procedures. Therefore, we use the less strict conditions in Equation (3) in order to reduce the type 1 errors, despite the increase in type 2 errors:

(3) $$\begin{array}{cc} 0{}^{\circ}\leq \text{H}\leq 55{}^{\circ}, & 300{}^{\circ}\leq H\leq 355{}^{\circ} \end{array}$$

**Figure 4.**
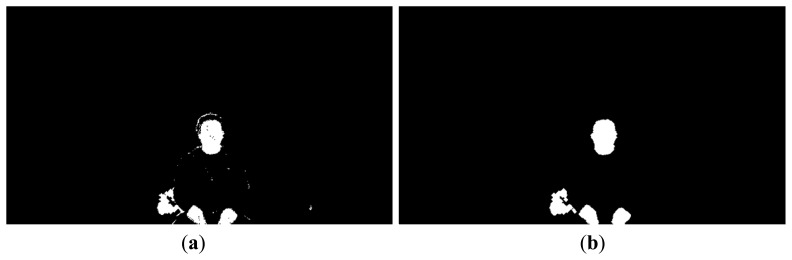
Color filtering examples: (**a**) Binary image after color filtering; (**b**) Resulting image after morphological operations.

**Figure 5.**
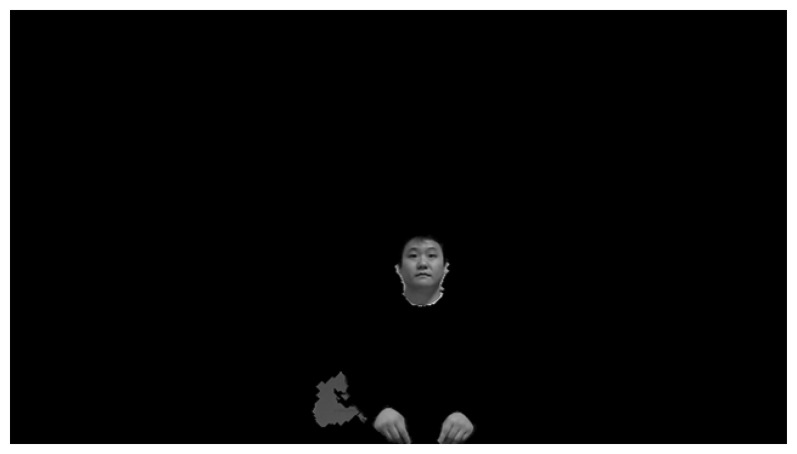
Example of the result from preprocessing by the client.

The results of color filtering are shown in Figure 4. Figure 4a,b shows the result after color filtering, and the resulting image after morphological operations that involve erosion and dilation, respectively. Figure 5 shows an example of the result of preprocessing on the client side. This image is sent to the server over the network.

### 2.4. Face Detection Robust to In-Plane Rotation

**Figure 6.**
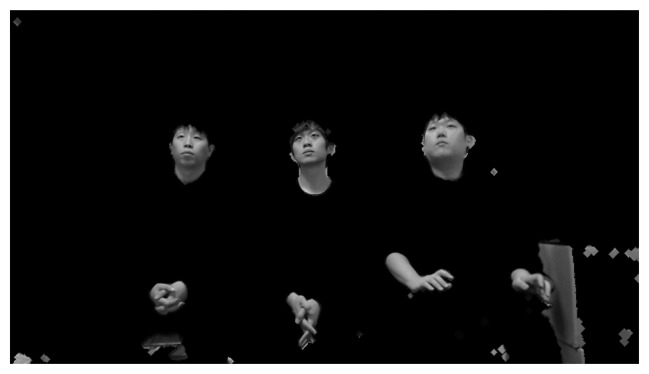
Preprocessed image that contains multiple rotated faces.

When the server receives the preprocessed images and face regions, these images may include multiple faces or rotated faces. This is because users can view various points on a TV while lying on their side. Figure 6 shows an example of a preprocessed image that contains rotated faces.

Image rotation and face detection are performed using the Adaboost method [10,11] in order to detect the face regions. In our research, we design the face detection & recognition system based on client & server structure, as shown in Figures 1 and 2, considering lots of clients (set-top boxes) connected to the server, which is often the case with the set-top box-based intelligent TV. Because lots of face candidates from many clients can be transmitted to the server at the same time and the final result of face recognition should be returned to the client at fast speed, more sophisticated algorithm of face detection which need high computation power is difficult to be used in the server although the server usually has higher processing power than client. The Adaboost-based face detection algorithm is one of the methods which are mostly used due to its high performance [16–18]. In addition, the Adaboost-based face detection method itself in the server is not the contribution of our research. Therefore, we used the face detection algorithm by Adaboost method in our research.

We reduce the number of cascades to 19 for the Adaboost face detector in order to increase the detection rate even though this results in an increase in the false positive detection rate. False positive detection means that a non-face area is incorrectly detected as a face area. Because a false positive face can be removed via further processing using GLDH and face recognition based on MLBP, we reduce the number of cascades to 19. The image rotation transform is as follows [9]:

(4) $$\left[\begin{array}{c} x'\\ y' \end{array}\right]=\left[\begin{array}{cc} \cos\theta & -\sin\theta \\ \sin\theta & \cos\theta \end{array}\right]\;\left[\begin{array}{c} x\\ y \end{array}\right]$$

where, *θ* is [−45°, −30°, −15°, 15°, 30°, 45°]. The origin for image rotation is the center of the image.

**Figure 7.**
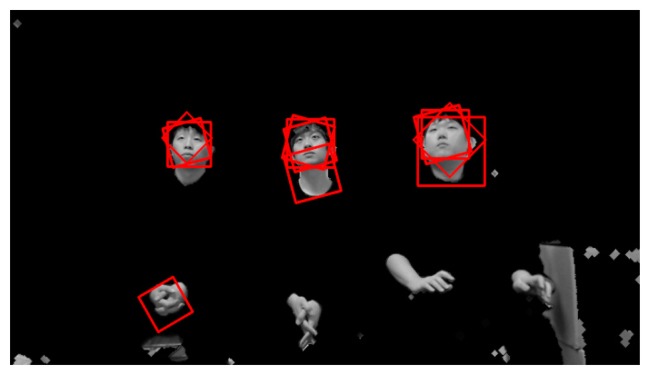
Multiple face boxes in the same face region.

Since six rotated images are obtained in addition to the original image, Adaboost face detection is performed seven times based on the face candidate regions in Figures 5 and 6. Thus, multiple face boxes can be produced even in the same face region, as shown in Figure 7. We use the GLDH method to choose the correct one from the multiple face boxes that are available because it can use the characteristics of face symmetry to estimate a vertical axis that optimally bisects the face region [12]. We can find the optimal face box based on the resulting vertical axis. However, the left half of the face area is not usually identical to the right half because of variations in illumination and face pose. As a result, it is not possible to estimate the similarity between the left and right sub-regions of a face based on simple pixel differences between these two sub-regions. Therefore, the GLDH method is employed as follows [12].

**Figure 8.**
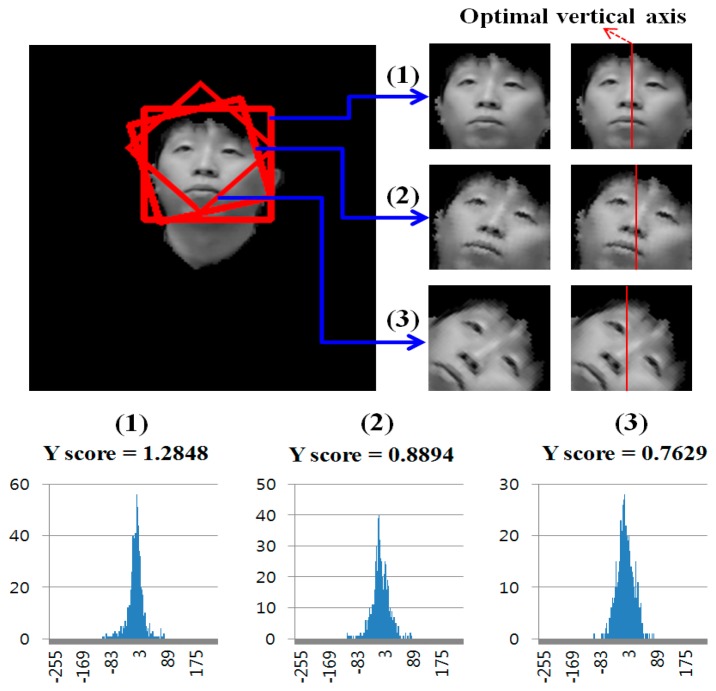
Examples of GLDH and Y scores.

We call the horizontal position of the vertical axis that evenly bisects the face box as the initial vertical axis position (IVAP). Then, the GLDHs are obtained at the five positions (IVAP – 10, IVAP – 5, IVAP, IVAP + 5, and IVAP + 10). The graphs of the GLDHs are shown at the bottom of Figure 8. The horizontal and vertical axes of the graphs show gray level difference (GLD) and the number (histogram) of the corresponding GLD, respectively [12].

The GLDHs are obtained at five positions because of the following reasons. If the detected face is rotated (yaw) in the horizontal direction, the IVAP is not the optimal axis for representing the symmetry. Therefore, we calculate the GLDH at five positions (IVAP – 10, IVAP – 5, IVAP, IVAP +5, and IVAP + 10). If one of the five positions leads to the optimal vertical axis, the corresponding GLDH distribution can show a sharp shape with a smaller level of variation. Based on this result, we can determine the correct face box by coping with the case where the detected face is rotated in the horizontal direction because the severe rotations of faces typically do not occur when users are watching TV.

We use the Y score defined in Equation (5) to measure the shape of the distribution [12]:

(5) $$Y\;\text{score}=\frac{\text{MEAN}}{\sigma^{2}}$$

The *MEAN* in Equation (5) is the number of pixel pairs whose GLD falls within a specified range (which we set at ±5) based on the mean of the distribution. The higher the *MEAN*, the more symmetric the axis is. The *σ* parameter represents the standard deviation of the distribution. The higher the Y score, the more symmetric the face region is based on the axis [12].

**Figure 9.**
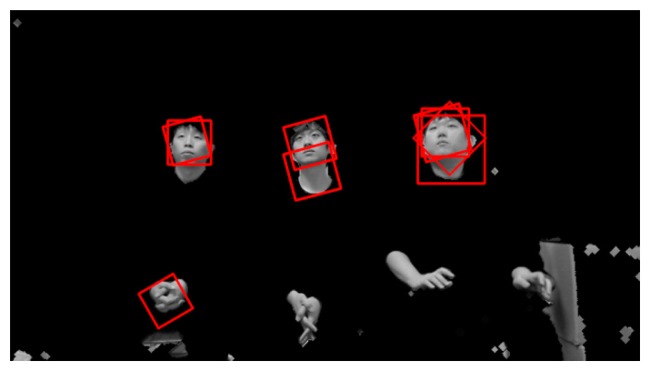
Chosen face boxes of Figure 7 using the GLDH method.

As shown in Figure 7, several faces are determined as belonging to the same facial group based on their distances from each other. That is, the face boxes whose inter-distances between centers are smaller than the threshold are designated as belonging to the same facial group. Then, the axes that have larger Y scores than the threshold are chosen from the facial group. Figure 8 shows the GLDH for the faces in one group from Figure 7 and their Y scores. Figure 9 shows the results for selected face boxes as determined on a per person basis by the GLDH method.

**Figure 10.**
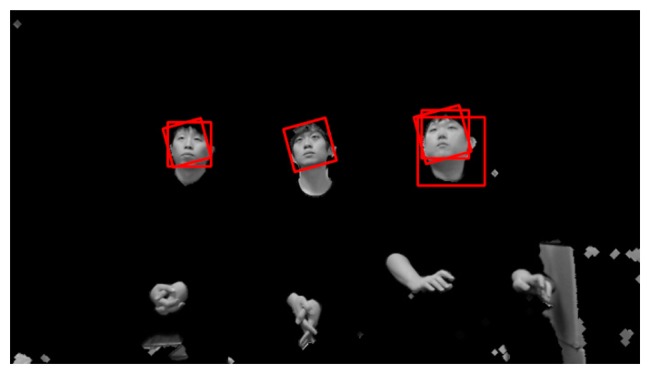
Results from face detection based on Figure 9 and the use of eye detection.

Eye regions are then detected in the face candidate regions based on the Adaboost eye detector [10,11]. If no eye regions are detected, the face region is regarded as a non-face region. Figure 10 shows the final results for face detection. Since it is often the case that users watch TV while lying on their sides, multiple rotated face boxes are used for face recognition in Section 2.5.

If the multiple face candidates from Figures 7 and 9 are used for face recognition, the processing time increases considerably. In addition, face recognition errors (false matching) increase because of the multiple trials during the recognition process.

### 2.5. Face Recognition Based on MLBP

**Figure 11.**
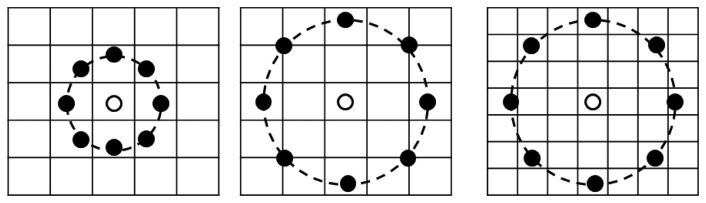
Multi-resolution LBP operators: *LBP*_8,1_, *LBP*_8,2_, and *LBP*_8,3_.

The detected face regions are used for face recognition. However, the detected face regions can still contain holes (*i.e.*, incorrectly rejected face pixels) because of image differences and color filtering and these holes can lead to face recognition errors. In order to rectify this, we interpolate the hole pixels in the detected face region. If a zero value pixel is found inside the detected region, it is compensated for by applying a 5 × 5 average mask and by excluding adjacent zero pixels. The interpolated face image is then normalized based on the two eye positions and used for face recognition based on the MLBP method. The basic operator for the local binary pattern (LBP) is a simple, yet powerful, texture descriptor. It is used for texture classification, segmentation, face detection, and face recognition [19]. The basic concept behind the LBP is the assignment of a binary code to each pixel based on a comparison between the center and its neighboring pixels. In order to acquire large-scale image structures, the basic LBP was extended to multi-resolution LBP, which is denoted as *LBP_P, R_*, as shown in Figure 11 [20,21]. By extending single-resolution LBP operator to a multi-resolution operator using the various *P* and *R* values, various (local and global) textures can be extracted for face recognition.

The *LBP_P, R_* code is obtained using Equation (6) [20,21]:

(6) $${\text{LBP}}_{P,R}=\sum_{p=0}^{P-1}s(g_{p}-g_{c})2^{p},\text{where}\;s(x)=\{\begin{array}{cc} 1, & x\geq 0\\ 0, & x<0 \end{array}$$

In this case, *P* is the number of neighboring pixels, and *R* is the distance between the center and the neighboring pixels, as shown in Figure 11. The *g_c_* parameter corresponds to the gray value of the center pixel. The *g_p_* parameter (where *p* = 1, …, *P*–1) are the gray values of the *p* that has equally spaced pixels on the circle of radius *R* that forms a circularly symmetric neighbor set. The *s*(*x*) function is the threshold function for *x* [20,21].

**Figure 12.**
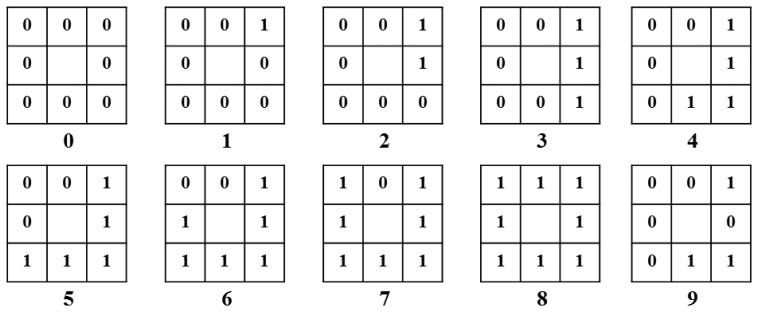
Examples of LBP codes for uniform patterns (0 to 8) and a non-uniform pattern (9) when *P* = 8, *R* = 1 from Figure 11.

The LBP codes are then divided into uniform and non-uniform patterns, as illustrated in Figure 12 [20,21]. A uniform pattern is a pattern that contains 0, 1, or 2 bitwise transitions from 0 to 1 (or 1 to 0), as shown in Figure 12. The others are called non-uniform patterns, as shown in pattern “9” in Figure 12. The uniform patterns can be used to detect spots, edges, or corners, whereas the non-uniform patterns do not contain sufficient information to represent a texture [21]. Thus, the same code can be assigned to all non-uniform patterns, as shown in pattern “9” in Figure 12. This leads to a reduction in the number of patterns.

We used *LBP*_16,3_. These values for *P* and *R* were experimentally determined as being optimal in terms of minimum errors during face recognition. The LBP operator obtained 18 texture patterns. The codes for the uniform patterns are numbered from zero to 16, and the non-uniform pattern is pattern 17.

**Figure 13.**
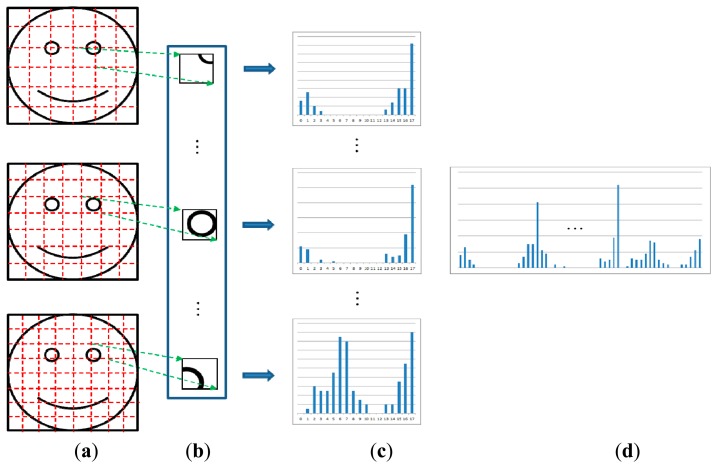
Extracting the histogram features using MLBP at three levels: (**a**) Face image that is divided into sub-blocks; (**b**) Sub-block regions; (**c**) Histograms for (b) created using LBP; (**d**) Final feature histogram obtained by concatenating the histograms from (c).

The final features for face recognition were obtained using the MLBP method [22]. Figure 13 shows the concept behind the MLBP feature extraction technique. The facial image is divided into sub-blocks, and the LBP histograms are calculated from each block as shown in Figure 13c. Because the number of texture patterns from the LBP operator is 18, we obtained a histogram with 18 components from each sub-block. In order to represent the histogram features globally and locally, the sub-blocks for faces are defined at three levels. The upper, middle, and lower faces in Figure 13a are the first, second, and third levels, respectively. That is, the global features are obtained from the sub-block for the face area for the first level. Local features are acquired from the sub-blocks for the third level because each sub-block is defined in the smaller region of the face. All of the histograms for each block are concatenated in order to form the final feature vector for face recognition, as shown in Figure 13d. We experimentally determined the optimal number of sub-blocks to be 6 × 6, 7 × 7, and 8 × 8 for the first, second, and third levels, respectively, in terms of minimum errors during face recognition.

We use the chi-square distance to measure the dissimilarity between enrolled face histogram features and the dissimilarity from the input image. The chi-square distance is expressed as follows in Equation (7) [23]:

(7) $$\chi^{2}(S,M)=\sum_{i}\frac{{\left(S_{i}-M_{i}\right)}^{2}}{S_{i}+M_{i}}$$

**Figure 14.**
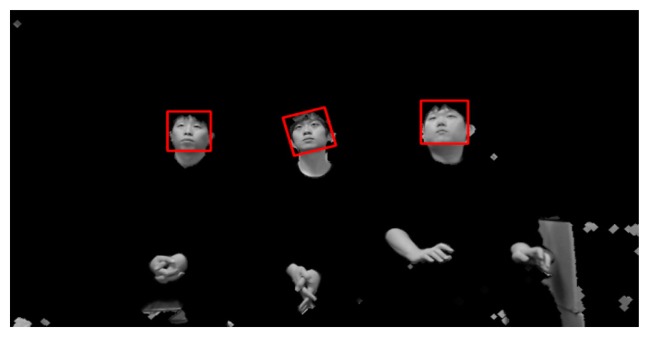
Examples of face regions that were finally recognized based on results from Figure 10.

Here, *S_i_* is the enrolled histogram and *M_i_* is the calculated histogram for the input image. In order to cope with faces in various poses (horizontal and vertical rotation), the face histogram feature for the input image is compared with the five enrolled ones (which were obtained at the initial enrollment stage) using Equation (7). The input face is then accepted as the enrolled person if the calculated distance is less than the predetermined threshold. The face region that has smallest chi-square distance in each facial group is determined as the finally recognized one as shown in Figure 14.

The reason why the histogram features in Figure 13 are used for face recognition, rather than the binary code with conventional LBP, is as follows. By using the histogram features, the rotation invariant code for the LBP can be generated without performing matching by bit-shifting. The rotation invariant uniform patterns are assigned to codes “0” to “8,” and non-uniform patterns are assigned to code “9,” as shown in Figure 12. Thus, the LBP histogram reflects the characteristics of rotation invariant textures for a bright spot, a dark spot, a uniform area, an edge, *etc.*, and non-textures. In addition, the process for matching based on the binary code from conventional LBP can be affected more significantly by the misalignment of the regions that are being compared than the process that depends on histogram features.

## 3. Experimental Results

We tested our proposed system on an STB (MIPS-based dual core 1.5 GHz, 1 GB DDR3 memory, and 256/512 MB NAND memory) that was connected to a web-camera and a server with a 3.5 GHz CPU and 8 GB of RAM. The experimental set-up of TV and the camera in Figure 1 was as follows: the size of the TV was 60 inch. The distance between the camera lens and the floor was about 79 cm. The distance between the TV (bottom of TV display) and the floor was about 84 cm.

**Figure 15.**
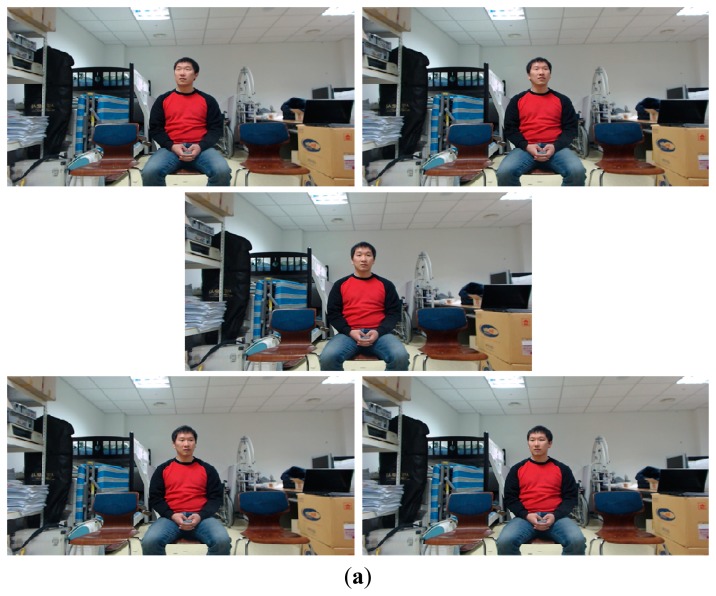

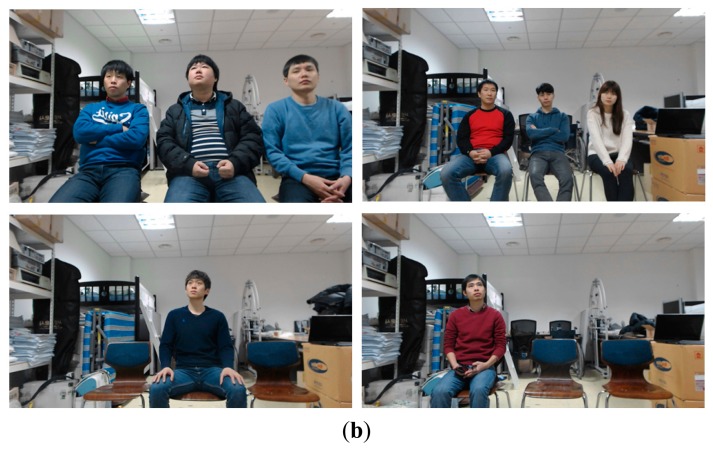
Examples of test images: (**a**) Examples of images for enrollment (where a user looks at top-left, top-right, middle, bottom-left, and bottom-right positions on the TV, respectively); (**b**) Examples of images for recognition.

Fifteen people, divided into five groups, participated in the test. The three persons in each group carried out five trials using the proposed system. The changes that were made between different trials included changes to the number of participants (one or three), changes to the seating positions (left, middle, right), and changes to the Z distances (1.5, 2, 2.5 m). Participants were instructed to look naturally and randomly at any point on the TV screen without restrictions. A total of 1350 frames (15 persons × 5 trials × 2 quantities of participants × 3 seating positions × 3 Z distances) were obtained for measuring the performance of the proposed system. In addition, a total of 75 frames (15 persons × 5 calibration points) were obtained for user calibration at the Z distance of 2 m. Figure 15 shows several examples of images from the experiments.

During the first experiment, we measured the accuracy of the face detection process with the 1350 images based on the recall and precision measures that are shown in Equations (8) and (9):

(8) $$\text{Recall}=\frac{N_{tp}}{M}$$

(9) $$\text{Precision}=\frac{N_{tp}}{N_{tp}+N_{fp}}$$

where *M* is the total number of faces in the images, *N_tp_* is the number of true positives, and *N_fp_* is the number of false positives. True positives mean that the faces were detected correctly, while false positives represent cases where non-faces were incorrectly detected as faces. If the recall value is close to 1, the accuracy of the face detection process is high. If the value of precision is 1, all of the detected face regions are correct with 0 false positives (*N_fp_* = 0).

We divided the images into five groups based on the 15 participants. Table 1 shows the experimental results based on the participant groups. As shown in Table 1, most of the values for recall and precision were similar, irrespective of the participant groups. The recall value for Group 2 was lower than the recall values for the other groups because the face detection failed because of the female with a hairstyle that occluded part of the face. In addition, the face size of user in Group 2 was comparatively small. As a result, the Adaboost method was unable to detect the user's face at 2.5 m. If we tried to change the parameters of Adaboost method in order to detect small faces, the number of incorrectly detected faces also increased in other cases. So, we did not change the parameter.

The precision values for Groups 2 and 3 were lower than those for other groups. The reason for this was that the participants were wearing clothing with colors that were similar to the color of facial skin and the number of false positives that resulted during the skin color filtering in Section 2.3 was relatively higher than for other groups.

In Table 2, we measured the face detection accuracies based on the Z distances of the participants. The recall values were lower when the participants were at the Z distance of 2.5 m. The reason for this is that the face sizes became smaller at the far Z distance and the face detection process failed as a result.

In Tables 3 and 4, we measured the face detection accuracies based on the seating positions and the number of participants, respectively. In order to calculate the precision value, *N_fp_* was measured as shown in Equation (9). False positives occur in the background area, but it is difficult to define the background region in cases where the results are based on the seating positions, as shown in Table 3. That is, when three participants are included in same image, it is difficult to separate the three backgrounds for the participants that are in the left, middle, and right seating positions. Therefore, in Table 3, the precision value was not measured. As shown in Tables 3 and 4, the face detection accuracies were similar irrespective of the seating positions and the number of participants.

During the second experiment, we measured the accuracy of the face recognition process based on genuine acceptance rate (GAR). By considering the use of the proposed system in a conventional family, we set the number of enrolled persons at 3. The GAR in Table 1 was calculated using only the face regions that were correctly detected, because the GAR measures the level of performance for face recognition, rather than detection.

As shown in Tables 1, 2, 3 and 4, we measured the GAR based on the participant groups, Z distances, seating positions, and number of participants in the input image. Since the images for enrolled participants were obtained at the Z distance of 2 m, the GAR at the 2 m was higher in Table 2 than it was in other cases. As shown in Tables 2, 3 and 4, we were able to confirm that the accuracy levels from our system were similar irrespective of the Z distances, seating positions, and the number of participants.

As the next experiment, we performed additional experiments in order to observe changes in the GAR based on the number of enrolled persons. We changed the number of enrolled persons to 4 and 5 from 3, and the GAR was calculated based on the number of enrolled persons as shown in Table 5. As shown in Table 5, the decrease in GAR was not significant as the number of enrolled persons increased.

If an incorrect face box was detected by the preprocessing in Figure 2a, or by the Adaboost face and eye detections, and during the face region verification by GLDH, then the face recognition process failed.

As the next test, we performed the comparative experiments with conventional methods [23–28] in the same results. Local binary pattern (LBP) is used for extracting the texture features in face [23] due to its superiority to illumination variation. In the conventional LBP method, the mask of 3 × 3 pixels is used to extract the texture feature. If the image of face region is obtained from the step (13) of Figure 2, the face image is normalized into that of 84 × 84 pixels.

With the face image of 84 × 84 pixels, we applied the LBP mask of 3 × 3 pixels. At each position of the LBP mask of 3 × 3 pixels in the face image, the surrounding eight pixel values of the face image is compared to the central one of the face image. If the surrounding pixel value is equal to or larger than the central one, 1 is assigned to the central position of the LBP mask at that position. If not, 0 is assigned. The number of positions (where the center of LBP mask can exist) is 82 × 82 in the face image of 84 × 84 pixels, and the number of bits obtained by LBP mask at each position is 8. Therefore, the total number of bits by the LBP method is 53,792 (82 × 82 × 8). Then, with these 53,792 binary bits, the dissimilarity between the two face images (enrolled and input ones) is calculated by hamming distance (HD). HD is calculated by the operation of exclusive OR (XOR). If two bits are same (“0” and “0”, or “1” and “1”), 0 is obtained by XOR. If two bits are different (“0” and “1”, or “1” and “0”), 1 is obtained. For example, if all the 53,792 bits extracted from the input face image are same to those from the enrolled one, the calculated HD is 0. If all the 53,792 bits from the input face image are completely different from those from the enrolled one, the calculated HD becomes 1. Our system finally selects the user of enrolled face bits (53,792 bits) (whose HD calculated with the input one is smallest among all the enrolled ones) as a genuine user.

Principal component analysis (PCA) is one of the most widely used method for face recognition by representing the facial features as global ones of eigen-coefficients based on eigenfaces [24,25]. Non-negative matrix factorization (NMF) is used for part-based representation by using non-negative values for the basis and coefficients [26]. Local non-negative matrix factorization (LNMF) is the enhanced method of NMF by making the local features more distinctive [26]. Support vector machine-discriminant analysis (SVM-DA) is the enhanced method of conventional linear discriminant analysis (LDA) based on the fusion of SVM and LDA by solving the limitation of LDA which assumes that all classes have same density function and Gaussian distributions [27]. Modified census transform (MCT) is the revised method of LBP by using the average value of the 3 × 3 pixels where the mask is applied instead of the central value of the 3 × 3 pixels [28]. Therefore, the number of bits obtained by the MCT mask at each position is 9 (as explained before, the number of bits is 8 in case of the LBP method). Because the number of positions (where the center of MCT mask can exist) is 82 × 82 in the face image of 84 × 84 pixels, the total number of bits by the MCT method is 60,516 (82 × 82 × 9). Then, with these 60,516 binary bits, the dissimilarity between the two face images (enrolled and input ones) is calculated by HD, also. Like the LBP method, our system finally selects the user of enrolled face bits (60,516 bits) (whose HD calculated with the input one is smallest among all the enrolled ones) as a genuine user. This kind of scheme (selecting the user of enrolled face features (whose distance calculated with the input one is smallest among all the enrolled ones) as a genuine user) is also used for other conventional methods of PCA, LNMF, SVM-DA, and our MLBP-based method.

The same data (which were used for the experiments of Table 5) were used for additional experiments. In detail, we did not apply the conventional methods (LBP, PCA, LNMF, SVM-DA, and MCT) directly to the input images. Instead, we applied them to the step of face recognition (step (14) of Figure 2). That is, the same steps (1)∼(13) of Figure 2 were used for both our MLBP-based method and the conventional ones, which means that we used the same face images (which were obtained through the steps (1)∼(13) of Figure 2) for both our method and the conventional ones for the fair comparisons.

As shown in Table 6, the GAR by our method is higher than previous ones. By extracting the histogram features using MLBP at three levels as shown in Figure 13, both global and local features for face recognition are efficiently obtained by our method, which causes the higher accuracy compared to previous ones.

As explained at the beginning part of Section 3, a total of 1350 frames (15 persons × 5 trials × 2 quantities of participants × 3 seating positions × 3 Z distances) were obtained for measuring the performance of the proposed system. The image size (resolution) of each people is reduced with the increase of Z distance, and the image data which were obtained at three different Z distances (1.5, 2, 2.5 m) include the effect of the change of image size (resolution), consequently.

As shown in Table 7, we show the face recognition accuracy (GAR) by our method compared to previous methods [23–28] according to the different Z distances. As shown in Table 7, the GARs by our method are higher than previous methods irrespective of the change of image resolution caused by the change of Z distance. By extracting the histogram features using MLBP at three levels as shown in Figure 13, both global and local features for face recognition are obtained by our method, which are less affected by the change of image resolution compared to previous ones.

**Figure 16.**
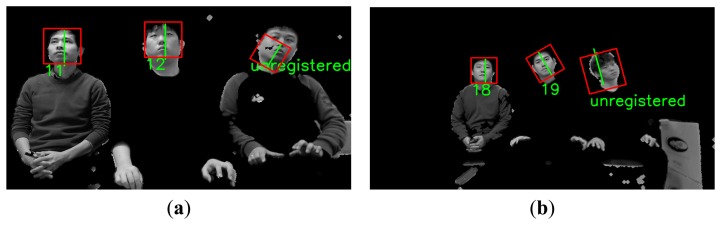
Examples of face recognition failures (**a**) Face recognition failed because of inaccurate preprocessing of Figure 2a; (**b**) Face recognition failed because of the incorrect detection of axis of face symmetry based on GLDH.

**Figure 17.**
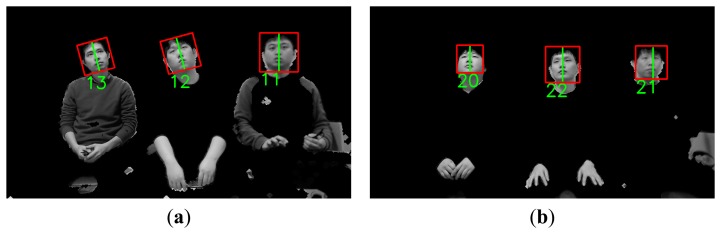
Examples of the success of face recognition.

Figure 16 shows examples where the proposed method failed during face recognition. Figure 17, on the other hand, shows examples where the proposed method succeeded during face recognition.

Experimental results showed that the processing time per each image is about 185 ms in our system. Therefore, our system can output result at the speed of about 5 or 6 frames per second. Considering our applications such as personalized advertizing and program recommendation services, child lock, and the automatic audience rating survey, our system having the processing speed of about 5 or 6 frames per second can be sufficiently used for these applications.

**Figure 18.**
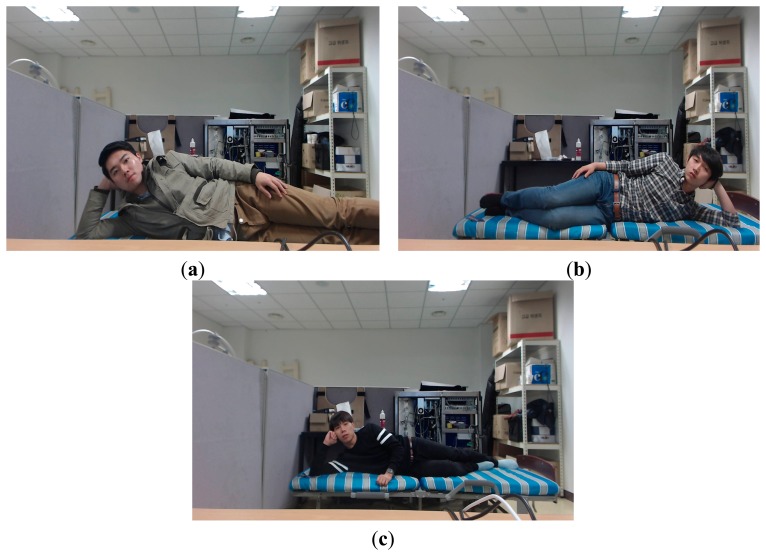
Examples of the images where each person is lying on his/her side. Image where a person is lying in the (**a**) left direction at the Z distance of 1.5 m; (**b**) right direction at the Z distance of 2 m; (**c**) left direction at the Z distance of 2.5 m.

As the next test, we performed additional experiment with the images where each person is lying on his/her side. A total of 300 images (5 persons × 3 Z distances (1.5, 2, 2.5 m) × 2 lying directions (left and right) × 10 images) were newly collected for experiment. Figure 18 shows the examples of the collected images. As shown in Table 8, the accuracies of face detection as recall and precision are 96.67% and 99.39%, respective, which are similar to the results of Tables 1, 2, 3 and 4. In addition, the accuracy of face recognition as GAR is about 93.1%, which is also similar to the results of Tables 1, 2, 3 and 4. From the Table 8, we can confirm that our system can be operated with the images of extremely rotated face.

## 4. Conclusions

In this paper, we proposed a new face recognition system for intelligent TVs with supplementary low resource STBs. Experimental results showed that recall, precision, and GAR values for the system were about 95.7%, 96.2%, and 90.2%, respectively. In addition, we confirmed that the accuracy of our system was similar, irrespective of the Z distances, seating positions, and the number of participants. However, if an incorrect face box was detected during preprocessing, during the Adaboost face and eye detections, or during the face region verification by GLDH, then face recognition failed.

Based on our face recognition results, we believe that our proposed system can provide personalized advertising services, log on services, and child lock services in conventional TVs with supplementary STBs. In a future study, we plan to test our system with a wider variety of STBs, servers, and network environments, including wired and wireless network environments.

## Figures and Tables

**Table 1.** Experimental Results based on Participant Groups (unit: %).

**Group**	**Recall**	**Precision**	**GAR**
1	96.85	98.87	90.76
2	89.44	91.15	93.2
3	97.78	92.63	82.89
4	95.74	98.86	96.98
5	98.7	99.26	87.33
Average	95.7	96.15	90.23

**Table 2.** Experimental Results based on the Z Distances (unit: %).

**Z Distance (m)**	**Recall**	**Precision**	**GAR**
1.5	100	96.81	89.11
2	95.78	96.59	92.97
2.5	91.33	96.47	88.61

**Table 3.** Experimental Results based on Seating Positions (unit: %).

**Seating Position**	**Recall**	**GAR**
Left	97.11	91.46
Middle	94.22	93.55
Right	95.78	85.64

**Table 4.** Experimental Results based on the Number of Participants (unit: %).

**The Number of Participants**	**Recall**	**Precision**	**GAR**
1	95.7	96.13	90.12
3	95.7	96.13	90.57

**Table 5.** GARs based on the number of enrolled persons (unit: %).

**Group**	**The Number of Enrolled Persons**		

	**3**	**4**	**5**
1	90.76	89.37	88.18
2	93.2	92.29	90.07
3	82.89	81.06	80.46
4	96.98	94.38	93.54
5	87.33	86.66	85.41
Average	90.23	88.75	87.53

**Table 6.** The comparisons of face recognition accuracy (GAR) of our method to previous ones (unit: %).

**Group**	**LBP**	**PCA**	**LNMF**	**SVM-DA**	**MCT**	**Proposed Method**
1	63.03	61.03	50.53	72.44	61.01	90.76
2	57.02	45.99	42.1	77.59	53.79	93.2
3	50.47	43.11	48.45	62.61	47.13	82.89
4	68.08	67.25	61.51	79.63	68.53	96.98
5	68.4	66.45	65.46	77.76	65.11	87.33
Average	61.4	56.77	53.61	74.01	59.11	90.23

**Table 7.** The comparisons of face recognition accuracy (GAR) of our method to previous ones according to different Z distances (unit: %).

**Z Distance (m)**	**LBP**	**PCA**	**LNMF**	**SVM-DA**	**MCT**	**Proposed Method**
1.5	63.06	53.51	52.71	76.55	58.59	89.11
2	64.96	57.16	56.02	79.29	63.78	92.97
2.5	56.18	59.4	52.1	66.17	54.98	88.61

**Table 8.** The accuracies of face detection and recognition with the images of extremely rotated face (unit: %).

**Recall**	**Precision**	**GAR**
96.67	99.39	93.1

## References

[1] Isobe T., Fujiwara M., Kaneta H., Uratani N., Morita T. (2003). Development and Features of a TV Navigation System. IEEE Trans. Consum. Electron..

[2] Zhang H., Zheng S., Yuan J. (2005). A Personalized TV Guide System Compliant with MHP. IEEE Trans. Consum. Electron..

[3] Zuo F., de With P.H.N. (2005). Real-time Embedded Face Recognition for Smart Home. IEEE Trans. Consum. Electron..

[4] An K.H., Chung M.J. (2009). Cognitive Face Analysis System for Future InteractiveTV. IEEE Trans. Consum. Electron..

[5] Lee S.-H., Sohn M.-K., Kim D.-J., Kim B., Kim H. Face Recognition of Near-infrared Images for Interactive Smart TV.

[6] Lin K.-H., Shiue D.-H., Chiu Y.-S., Tsai W.-H., Jang F.-J., Chen J.-S. Design and Implementation of Face Recognition-aided IPTV Adaptive Group Recommendation System Based on NLMS Algorithm.

[7] Lee S.-H., Sohn M.-K., Kim D.-J., Kim B., Kim H. Smart TV Interaction System Using Face and Hand Gesture Recognition.

[8] Logitech BCC950 http://www.logitech.com/en-us/support/conferencecam?section=overview&crid=637&osid=14&bit=64.

[9] Gonzalez R.C., Woods R.E. (2002). Digital Image Processing.

[10] Viola P., Jones M. Rapid Object Detection Using a Boosted Cascade of Simple Features.

[11] Viola P., Jones M.J. (2004). Robust Real-time Face Detection. Int. J. Comput. Vis..

[12] Chen X., Rynn P.J., Bowyer K.W. Fully Automated Facial Symmetry Axis Detection in Frontal Color Images.

[13] Phung S.L., Bouzerdoum A., Chai D. A Novel Skin Color Model in YCbCr Color Space and Its Application to Human Face Detection.

[14] Wang Y., Yuan B. (2001). A Novel Approach for Human Face Detection from Color Images under Complex Background. Pattern Recognit..

[15] Zhang X.-N., Jiang J., Liang Z.-H., Liu C.-L. (2010). Skin Color Enhancement Based on Favorite Skin Color in HSV Color Space. IEEE Trans. Consum. Electron..

[16] Xu Y., Fang X., Li X., Yang J., You J., Liu H., Teng S. (2014). Data uncertainty in face recognition. IEEE Trans. Cybern..

[17] Li S.Z., Chu R., Liao S., Zhang L. (2007). Illumination invariant face recognition using near-infrared images. IEEE Trans. Pattern Anal. Mach. Intell..

[18] Bhatt H.S., Singh R., Vatsa M. (2014). On recognizing faces in videos using clustering-based re-ranking and fusion. IEEE Trans. Inf. Forensic Secur..

[19] Ojala T., Pietikäinen M., Harwood D. (1996). A Comparative Study of Texture Measures with Classification Based on Feature Distributions. Pattern Recognit..

[20] Ojala T., Pietikäinen M., Mäenpää T. (2002). Multiresolution Gray-scale and Rotation Invariant Texture Classification with Local Binary Patterns. IEEE Trans. Pattern Anal. Mach. Intell..

[21] Choi S.E., Lee Y.J., Lee S.J., Park K.R., Kim J. (2011). Age Estimation Using a Hierarchical Classifier Based on Global and Local Facial Features. Pattern Recognit..

[22] Happy S.L., Georage A., Routray A. A Real Time Facial Expression Classification System Using Local Binary Patterns.

[23] Ahonen T., Hadid A., Pietikäinen M. Face Recognition with Local Binary Patterns.

[24] Belhumeur P.N., Hespanha J.P., Kriegman D.J. (1997). Eigenfaces *vs.* fisherfaces: Recognition using class specific linear projection. IEEE Trans. Pattern Anal. Mach. Intell..

[25] Turk M., Pentland A. (1991). Eigenfaces for recognition. J. Cogn. Neurosci..

[26] Li S.Z., Hou X.W., Zhang H.J., Cheng Q.S. Learning Spatially Localized, Parts-based Representation.

[27] Kim S.-K., Park Y.J., Toh K.-A., Lee S. (2010). SVM-based feature extraction for face recognition. Pattern Recognit..

[28] Froba B., Ernst A. Face Detection with the Modified Census Transform.

